# Randomised Controlled Trial Comparing Single Maintenance and Reliever Therapy (SMART) with Inhaled Budesonide-Formoterol Combination, *Versus* Conventional Budesonide and Additional As-Needed Levo-Salbutamol, in Children with Persistent Bronchial Asthma: Correspondence

**DOI:** 10.1007/s12098-026-06203-y

**Published:** 2026-06-03

**Authors:** Hongxiao Gao, Tongyuan Zhang

**Affiliations:** Tianjin University of Traditional Chinese Medicine, Tianjin, 301617 China

*To the Editor*: We read with interest the randomized trial by Basson et al. [1], which evaluated single maintenance and reliever therapy (SMART) against conventional budesonide with as-needed levo-salbutamol in children aged 6–11 y with persistent asthma. The study addresses an important question, as pediatric evidence for SMART remains limited.

One issue that may deserve closer attention is whether some of the observed advantage of SMART was related to regimen simplicity and better adherence rather than to pharmacologic superiority alone. Adherence was already higher in the SMART group at 4 wk and remained numerically higher thereafter [1]. This is relevant because simpler inhaled regimens and fewer daily treatment steps may improve adherence in children, and adherence itself can materially influence asthma control [2, 3].

The weekly symptom score also incorporated rescue-medication use [1]. Because the reliever strategy differed by design between groups, part of the between-group difference may have reflected treatment burden as well as biological effect. A brief analysis of the contribution of adherence and reliever use would help place the findings in context.

Future pediatric trials may therefore benefit from objective adherence monitoring and comparator regimens more closely matched for device complexity and dosing burden [2–4]. We congratulate the authors for providing valuable pediatric data; clarification of this aspect could further define the place of SMART in children aged 6–11 y.
